# Analysis of diverting ileostomy for colorectal cancer surgery: stenosis and T4 invasion are risk factors of reoperation due to anastomotic leakage, even when ileostomy is performed

**DOI:** 10.1007/s00384-026-05124-8

**Published:** 2026-03-30

**Authors:** Kohki Takeda, Takeshi Yamada, Ryo Ohta, Kay Uehara, Akihisa Matsuda, Seiichi Shinji, Yasuyuki Yokoyama, Goro Takahashi, Takuma Iwai, Koki Hayashi, Hiroshi Yoshida

**Affiliations:** Department of Gastroenterological Surgery, Nippon Medical School Hospital, 1-1-5 Sendagi, Bunkyo-Ku, Tokyo, 113-8603 Japan

**Keywords:** Colorectal cancer, Diverting ileostomy, Anastomotic leakage, Stoma-related complication

## Abstract

**Purpose:**

In colorectal cancer (CRC), diverting ileostomy prevents the occurrence and severity of anastomotic leakage (AL) during surgery. However, an ileostomy cannot prevent reoperation due to severe AL in some cases, and an approach other than ileostomy may be required. This study identified the risk factors of AL and reoperation due to AL in patients with diverting ileostomies.

**Methods:**

Patients diagnosed with CRC who underwent resection surgery accompanied by diverting ileostomy between January 2015 and December 2023 were included. We analyzed the risk factors for AL and reoperation due to AL. Stoma-related complications and perioperative results of stoma reversal surgery were also analyzed.

**Results:**

In total, 120 patients were enrolled. AL occurred in 21 (17.5%) patients. Multivariate analysis revealed that tumor location in the lower rectum was the only risk factor for AL (*P* = 0.0095). Of these 21 patients, four (19.0%) required reoperation, while 17 (81.0%) recovered without reoperation. The rates of T4 tumors (*P* = 0.022) and stenosis (P < 0.001) were significantly higher in the reoperation group. Among the 120 patients, a high-output stoma was observed in 36 patients (30.0%), and outlet obstruction occurred in 19 patients (15.8%). In stoma reversal surgery, two patients (1.7%) experienced severe complications (Clavien–Dindo grade ≥ III).

**Conclusion:**

Lower rectal tumors are associated with a high risk of AL, and diverting ileostomy should be considered in such cases. Due to small number of AL patients requiring reoperation, the finding is exploratory. However, in patients with stenosis and T4 invasion, the merits of ileostomy might be restricted.

**Supplementary Information:**

The online version contains supplementary material available at 10.1007/s00384-026-05124-8.

## Purpose

The safety of colorectal cancer (CRC) surgery has significantly improved over the last few decades. However, a certain number of perioperative complications were inevitable. Especially, anastomotic leakage (AL) is a major complication, leading to increased morbidity rate and rate of local and distant recurrence [1–4]. Rabhari et al. [5] defined three AL categories. Grade A: leaks do not require any intervention; Grade B: leaks require active intervention but without relaparotomy; and Grade C: leaks require relaparotomy or relaparoscopy.

To prevent the occurrence and severity of AL, diverting stomas have been performed, and two recent meta-analyses have shown a lower rate of AL in patients receiving diverting stomas [6, 7]. Loop ileostomy and transverse colostomy are two common diverting stomas. The advantages and disadvantages of both stomas have been compared in several studies, and some studies recommend the use of ileostomies [8–10].

However, ileostomy is associated with several stoma-related complications such as high output stoma (HOS) and stoma-related small bowel obstruction (SBO) [11]. Stoma reversal surgery (ileostomy closure) is disadvantageous. Therefore, ileostomy should only be performed in necessary cases. Furthermore, although an ileostomy is performed, reoperation due to severe AL cannot be prevented in some cases. In such cases, a different approach to ileostomy is required.

In the present study, patients with CRC who underwent diverting ileostomy were analyzed. This study aimed to identify the risk factors of AL and reoperation due to AL. Stoma-related complications and perioperative results of stoma reversal surgery were also analyzed.

## Methods

### Patients

This single-institution, retrospective study was approved by the Ethics Committee of Nippon Medical School (Tokyo, Japan, M-2021–029). Patients diagnosed with CRC who underwent resection surgery accompanied by diverting ileostomy between January 2015 and December 2023 were included. Only elective surgeries were included, and emergency surgeries were excluded.

Regarding patient characteristics, the previous treatment history included chemotherapy and/or radiation therapy. Stenosis was defined as a condition in which the colonoscope could not pass through. Patients in whom the colonic stent was replaced and the colonoscope became able to pass through were defined as having no stenosis. The depth of cancer invasion, the presence of lymph node metastasis, and cancer stage were based on the pathological findings of the resected tumor, including patients who had received previous treatment. Cancer stage was based on the 8th edition of the Tumor, Node, and Metastasis (TNM) classification.

### Criteria and method of diverting ileostomy construction

There were no definite criteria for ileostomy construction. According to past studies, patients with a high risk of AL, such as male sex, obesity, many comorbidities (diabetes, renal failure, etc.), with previous treatment history, stenosis, and tumors in the lower rectum were candidates for ileostomy construction. The final decision was made by the operator.

In all the patients, the stoma site was marked before surgery. The ileum, located approximately 40 cm from the terminal ileum, was raised without torsion. The ileum was not fixed to the anterior sheath of the rectus abdominis. An incision was made in the raised ileum to create double orifices, which were fixed to the skin using 12–16, 3–0 or 4–0 absorbable sutures.

### Diagnosis of AL and indication of reoperation

AL was diagnosed based on the clinical and imaging findings. Clinical findings included pain, fever, and enteric drainage. Imaging findings included fluid- or gas-containing collections revealed on Computed Tomography (CT). The indication for reoperation was based on the presence of pan-peritonitis (peritoneal irritation symptoms spreading to a wide abdominal area) and vital sign disorders, and the decision was made by the attending physician.

### Diagnosis and definition of HOS and outlet obstruction

This study defined HOS as a stoma output of 1500 ml/24 h. SBO at the stoma site is often referred to as an outlet obstruction. The diagnosis of outlet obstruction was made based on CT findings, which revealed small intestinal dilation caused by the site where the abdominal wall had penetrated.

### Method of ileostomy closure

In all patients, the anastomosis was checked by colonoscopy before ileostomy closure. A skin incision was made around the stoma. The ileostomy was dissected from the rectus sheath and peritoneal cavity. The ileum was resected, followed by a stapled, functional end-to-end anastomosis. No drainage tubes were inserted.

### Risk factors of AL and reoperation due to AL

Patients were divided into two groups [1]. AL group (patients with confirmed AL) and [2] non-AL group (patients without confirmed AL). The AL and non-AL groups were compared to evaluate predictive risk factors for AL. Eleven factors were identified using univariate and multivariate analyses. The 11 factors were age, sex, body mass index (BMI), history of diabetes (DM), Charlson comorbidity index (CCI), previous treatment history (chemotherapy and/or radiation therapy), presence of stenosis, tumor location (lower rectum or not), depth of invasion (T4 or not), presence of lymph node metastasis, and presence of distant metastases.

Furthermore, the AL group was divided into two groups [3]. The reoperation group (patients who underwent reoperation due to AL) and the conservative treatment group (patients who recovered without reoperation) [4]. This study compared the reoperation and conservative treatment groups to evaluate predictive risk factors for reoperation. Twelve factors were identified (the same 11 factors and position of the drainage tube) using univariate analysis.

### Analysis of stoma-related complications and surgical outcomes of ileostomy closure

The occurrence rates and outcomes of HOS and outlet obstruction were analyzed in all patients. This study also analyzed the surgical outcomes of ileostomy closure. The primary endpoint was severe complications, defined as Clavien–Dindo (CD) grade ≥ III. Secondary outcomes included duration of surgery, intraoperative bleeding volume, and any complication: CD grade ≥ I.

### Statistical analysis

All statistical analyses were performed using EZR version 1.66 (Saitama Medical Center, Jichi Medical University, Saitama, Japan), a graphical user interface for R version 3.0.2 (R Foundation for Statistical Computing, Vienna, Austria). Comparisons were performed using Pearson’s chi-squared test for categorical variables and the Mann–Whitney U test for quantitative variables. Statistical significance was set at P < 0.05, which was considered significant.

We used the STROBE reporting guideline [12] to draft this manuscript, and the STROBE reporting checklist [13] when editing, included in the supplementary material.

## Results

### Patients

During the study period, 2388 patients with CRC underwent resection surgery. Among these patients, 120 patients (5.0%) underwent surgery accompanied by diverting ileostomy. Ninety (75.0%) patients were male, 45 (37.5%) had received previous treatment, and 14 (11.7%) had stenosis. In four patients, colonic stent was replaced. In all these four patients, the colonoscope became able to pass through, therefore were classified as having no stenosis. The tumor location was Rb in 60 patients (50.0%), and the depth of invasion was T4 in 12 patients (10.0%). Patient characteristics are summarized in Table 1.

**Table 1:** Patient characteristics

		*N* = 120
Sex (male/female)		90/30
Age		67 (27–86)
BMI		22.6 (16.7–35.3)
CCI		0.48 (0–3)
Previous treatment history (yes/no)		45/75
Stenosis (yes/no)		14/106
Tumor location	Transverse colon	1
	Descending colon	1
	Sigmoid colon	5
	Upper rectum	53
	Lower rectum	60
Depth of invasion	T0	2
	Tis	3
	T1	12
	T2	32
	T3	59
	T4	12
Stage	0	4
	1	35
	2	27
	3	49
	4	5

Data on age and BMI are shown as medians (ranges), and CCI data are shown as averages (ranges)

*BMI*, body mass index, *CCI* Charlson comorbidity index

**Fig. 1:**
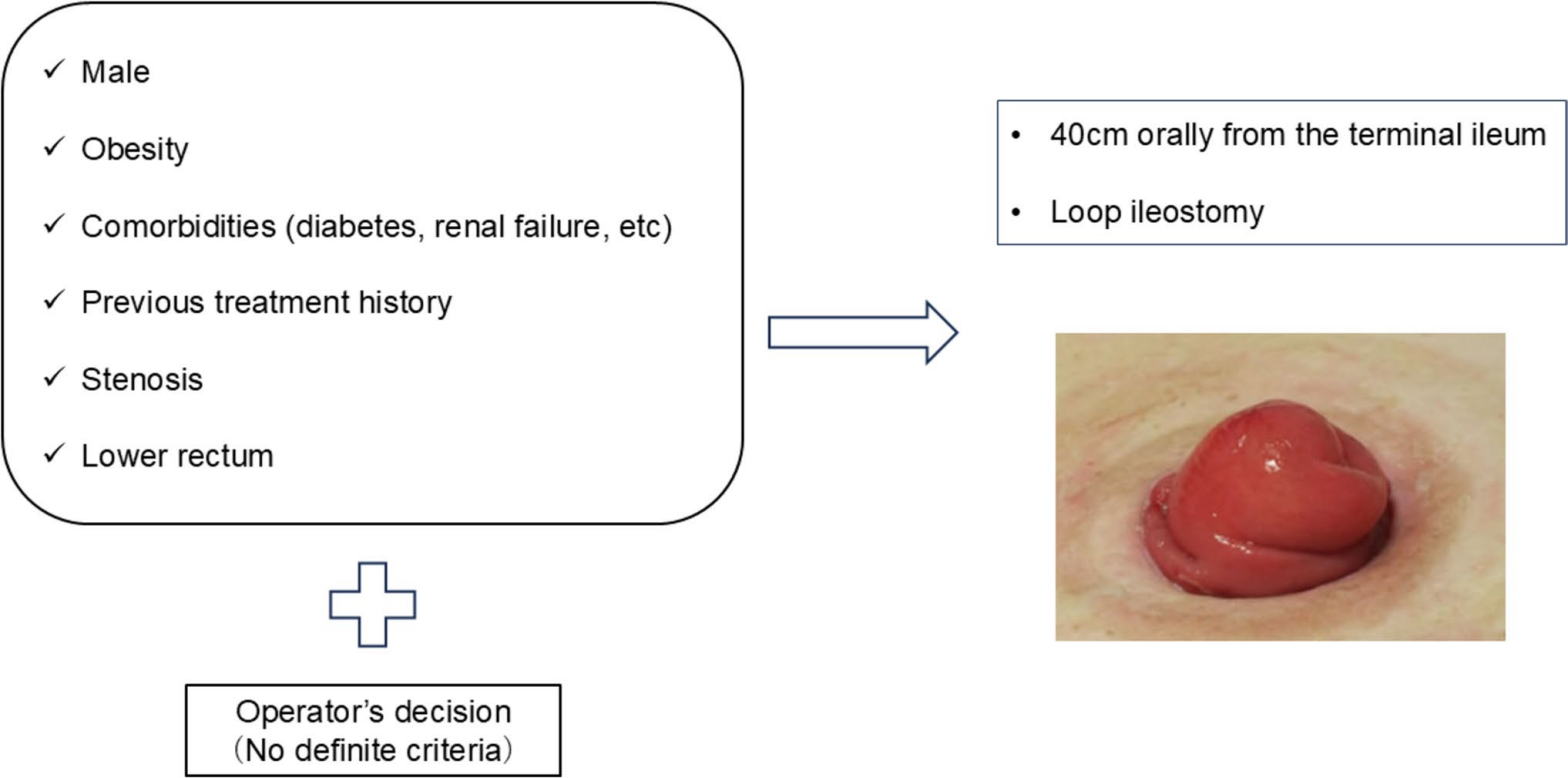
Image of covering ileostomy in our department

**Fig. 2:**
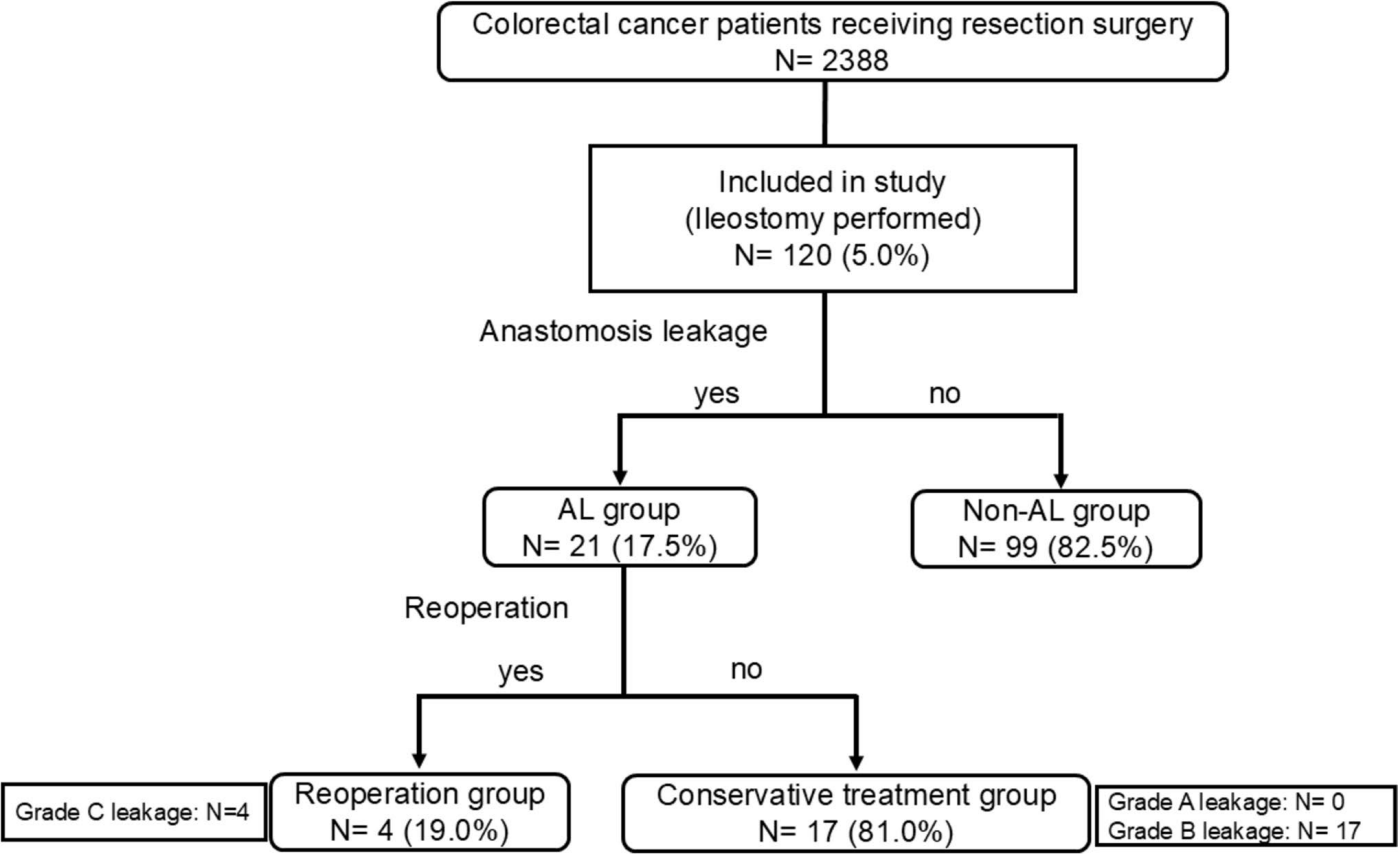
Patient selection flow diagram

Among the 120 patients included in this study, AL occurred in 21 (17.5%) patients (AL group: *N* = 21, non-AL group: *N* = 99). Of these 21 patients, four (19.0%) needed reoperation (Grade C leakage), while 17 (81.0%) recovered without reoperation (reoperation group, *N* = 4; conservative treatment group, *N* = 17). Of the 17 patients in the conservative treatment group, all 17 patients were grade B leakage, and no patients were grade A leakage. Figure 1 shows an image of the covering ileostomy performed in our department. A patient selection flow diagram is shown in Fig. 2.

### Risk factors of AL and reoperation due to AL

Univariate analyses of the 11 factors in the AL (*N* = 21) and non-AL (*N* = 99) groups are summarized in Table 2. The rate of tumor location in the lower rectum was significantly higher (*P* = 0.0018) in the AL group (81.0%) than in the non-AL group (43.4%). The other ten factors showed no significant differences. Multivariate analysis (Table 3) showed that tumor location in the lower rectum was the only independent risk factor for AL (*P* = 0.0095).

**Table 2:** Comparison between AL and non-AL groups assessed using univariate analysis (risk factors for AL)

	AL group (*N* = 21)	Non-AL group (*N* = 99)	*P* value
Age	63 (49–78)	67 (27–86)	0.26
Sex (male)	18 (85.7%)	72 (72.7%)	0.21
BMI	22.1 (18.3–30.8)	22.6 (16.7–35.3)	0.30
DM (yes)	4 (19.0%)	23 (23.3%)	0.68
CCI	0.48 (0–3)	0.48 (0–3)	0.61
Previous treatment history (yes)	9 (42.9%)	36 (36.7%)	0.58
Stenosis (yes)	3 (14.3%)	11 (11.1%)	0.68
Tumor location (Rb)	17 (81.0%)	43 (43.4%)	0.0018
Depth of invasion (T4≦)	2 (9.5%)	10 (10.1%)	0.94
Lymph node metastasis (yes)	11 (52.4%)	41 (41.4%)	0.36
Distant metastasis (yes)	1 (4.8%)	4 (4.0%)	0.88

Data on age and BMI are shown as medians (ranges), and CCI data are shown as averages (ranges)

*AL* anastomotic leakage, *BMI* body mass index, *DM* diabetes, *CCI* Charlson Comorbidity Index

**Table 3:** Multivariate analysis of independent risk factors for AL

	Odds ratio	95% CI	P value
Age (75 ≤)	0.31	0.05–2.03	0.22
Sex (male)	2.86	0.65–12.53	0.16
BMI (22.53 ≤)	0.29	0.14–1.34	0.15
DM (yes)	0.88	0.13–5.89	0.89
CCI (1 ≤)	0.58	0.11–3.12	0.52
Previous treatment history (yes)	0.86	0.26–2.82	0.80
Stenosis (yes)	4.62	0.22–99.15	0.33
Tumor location (Rb)	8.63	1.69–44.03	0.0095
Depth of invasion (T4 ≤)	1.46	0.05–45.93	0.83
Lymph node metastasis (yes)	1.34	0.45–4.00	0.60
Distant metastasis (yes)	2.57	0.17–39.49	0.50

*AL* anastomotic leakage, *CI* confidence interval, *BMI* body mass index, *DM* diabetes, *CCI* Charlson Comorbidity Index

Univariate analyses of the 12 factors in the reoperation (*N* = 4) and conservative treatment (*N* = 17) groups are summarized in Table 4. The drainage tube position was adequate for all patients in both groups. The rates of T4 tumors and stenosis were significantly higher (T4, *P* = 0.0022; stenosis, P < 0.001) in the reoperation group (T4,50.0%; stenosis, 75.0%) than in the conservative treatment group (T4,0%; stenosis, 0%).

**Table 4:** Comparison between the reoperation and conservative treatment groups was performed using univariate analysis (risk factors for reoperation)

	Reoperation group (*N* = 4)	Conservative treatment group (*N* = 17)	*P* value
Age	66 (53–74)	62 (49–78)	0.65
Sex (male)	4 (100%)	14 (82.4%)	0.36
BMI	21.3 (18.3–30.8)	22.1 (18.4–28.6)	0.79
DM (yes)	1 (25.0%)	3 (14.3%)	0.74
CCI	0.74 (0–3)	0.41 (0–2)	0.61
Previous treatment history (yes)	3 (75.0%)	6 (35.3%)	0.15
Stenosis (yes)	3 (75.0%)	0 (0%)	< 0.001
Tumor location (Rb)	2 (50.0%)	15 (88.2%)	0.08
Depth of invasion (T4 ≤)	2 (50.0%)	0 (0%)	0.0022
Lymph node metastasis (yes)	2 (50.0%)	9 (52.9%)	0.92
Distant metastasis (yes)	0 (0%)	1 (5.9%)	0.62
Position of the drainage tube (adequate)	4 (100%)	17 (100%)	1.00

Data on age and BMI are shown as medians (ranges), and CCI data are shown as averages (ranges)

*BMI* body mass index, *DM* diabetes, *CCI* Charlson Comorbidity Index

### Analysis of stoma-related complications and surgical outcomes of ileostomy closure

Among the 120 patients included in this study, HOS was observed in 36 (30.0%) patients. Of these 36 patients, HOS was successfully treated by oral medicine in 34 patients (94.4%). In two patients (5.6%), HOS and dehydration were difficult to treat; therefore, early ileostomy closure was performed.

**Fig. 3:**
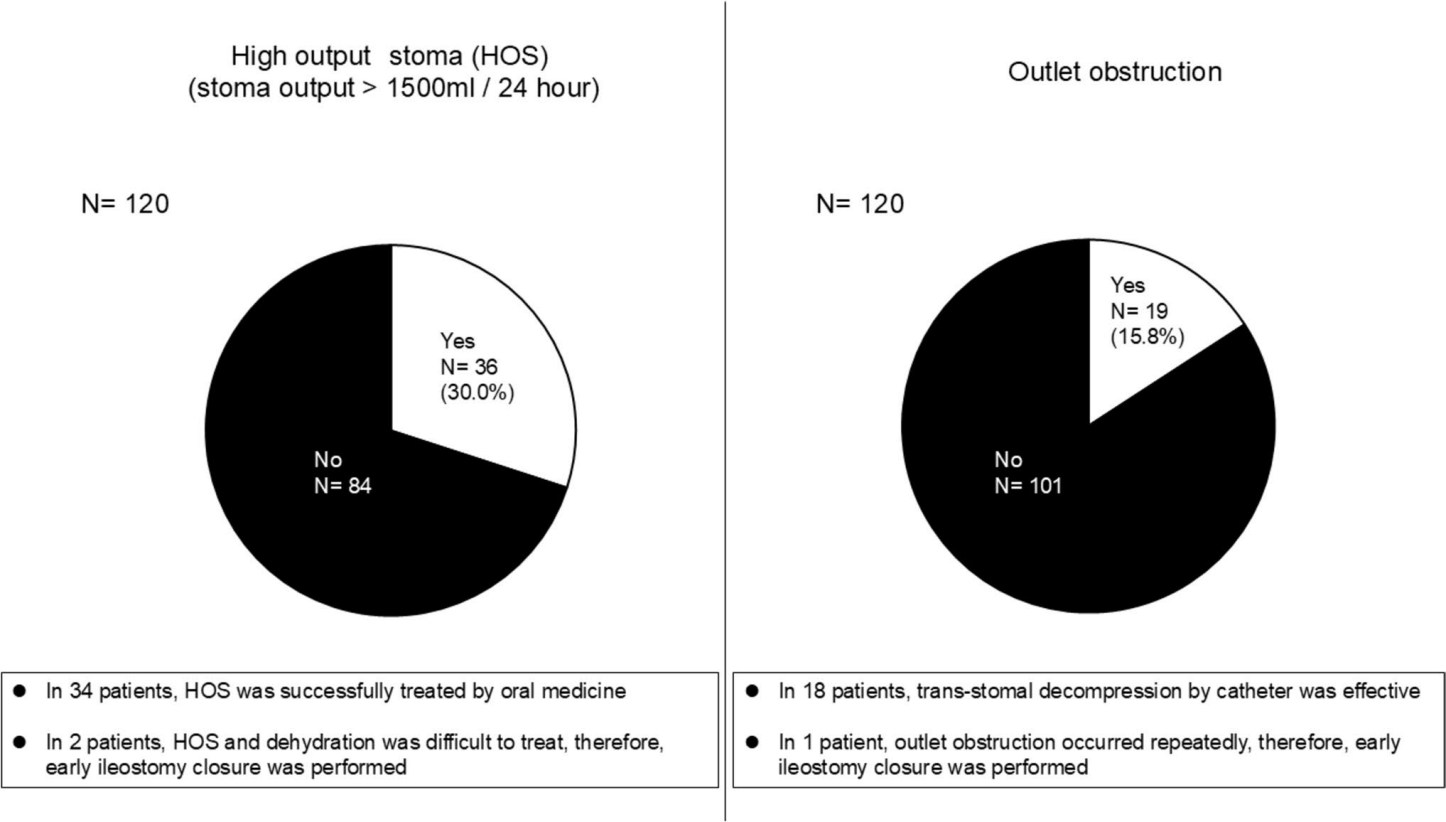
Results of stoma-related complications

Outlet obstruction occurred in 19 (15.8%) patients. Transtomal decompression using a catheter was effective in 18 patients (94.7%). In one patient (5.3%), outlet obstruction occurred repeatedly; therefore, early ileostomy closure was performed. The results of stoma-related complications are shown in Fig. 3.

All 120 patients received ileostomy closure. The surgical outcomes of the ileostomy closures are summarized in Table 5. The median time between stoma construction and closure was 141 days. Among the 120 patients, two patients (1.7%) experienced severe complications: C–D grade ≥ III. One patient had stage IV disease.

**Table 5:** Surgical outcomes of ileostomy closure

*N* = 120	
Time of stoma construction to closure (days)	141 (12–373)
Operation time (minutes)	88 (42–209)
Intraoperative bleeding volume (ml)	17 (0–384)
Followed period after ileostomy closure (months)	44 (6–99)
Postoperative complications	
CD grade ≧ I	10 (8.3%)
CD grade ≧ III	2 (1.7%)

Data are shown as medians (ranges)

*CD* Clavien–Dindo

with severe postoperative pneumonia. In another patient, the AL of the rectal anastomosis relapsed after stoma closure. Ten patients (8.3%) experienced postoperative complications of any type: C–D grade ≥ I. No cases revealed AL of the ileum anastomosis. The median followed period after ileostomy closure was 44 months.

## Discussion

This study presents three novel and valuable findings. First, in patients with CRC receiving resection surgery accompanied by diverting ileostomy, tumor location in the lower rectum was the only independent risk factor for AL. Second, stenosis and T4 invasion were risk factors for reoperation due to AL, even though an ileostomy was performed. Third, the surgical outcomes of the ileostomy closure were favorable.

In the present study, tumor location in the lower rectum was the only independent risk factor for AL. Previous studies have reported several risk factors for AL, such as the distance of the anastomosis from the anal verge, male sex, DM, obesity, preoperative radiochemotherapy, weight loss, and combined multiorgan resection [14–17]. However, most studies have analyzed the risk factors of AL during colorectal surgery without diverting stomas. The present study is unique because it focused on patients with a high potential for AL and analyzed the risk factors for AL only in patients who underwent diverting ileostomy. Interestingly, tumor location was the only risk factor in patients with a high potential for AL, and this finding may lead to the avoidance of unnecessary ileostomy.

Reoperation due to AL frequently occurs in patients with stenosis and T4 invasion. In a meta-analysis by Ang et al., the reoperation rate in patients with AL was 75.2% in patients without diverting stomas and 39.0% in patients with diverting stomas. This meta-analysis did not consider the risk factors for reoperation [18]. In this present study, the reoperation rate was 19.0%. Stenosis and T4 invasion were risk factors in the univariate analysis. Multivariate analysis was not performed due to the small number of AL cases. However, this result is reasonable for actual clinical situations. In patients with stenosis and T4 invasion, the amount of fecal retention between the ileostomy and anastomosis is large, and the efficiency of the ileostomy is thought to be limited. In these cases, an alternative strategy to ileostomy, such as colostomy, prolonged fasting period before the operation, and enhanced bowel preparation, might be required.

The perioperative result of ileostomy closure was favorable. Among the 120 patients, only 2two patients (1.7%) experienced severe complications: C–D grade ≥ III. None of the patients had an AL in their ileal anastomosis. In the meta-analysis published in 2021, the rate of AL of the ileum anastomosis was 3.5%, and the rate of severe complications: C–D grade ≥ III was 3.6% [19], similar to the present study. Conversely, the rate of stoma-related complications was relatively low. In this study, HOS occurred in 36 patients (30.0%), and outlet obstruction occurred in 19 patients (15.8%). According to previous reports, the incidence of HOS and outlet obstruction was approximately 16% [20, 21]and 23% [22, 23], respectively. However, HOS was successfully treated with oral medicine, and the outlet obstruction was controlled by transstomal decompression in most patients. Considering the benefits of ileostomy in preventing the occurrence and severity of AL, and the favorable surgical outcomes of ileostomy closure, ileostomy construction is recommended in cases where it is difficult to decide whether ileostomy is necessary, especially in cases involving the lower rectum.

This study had some limitations. First, it was a retrospective study conducted at a single institution. Second, only four patients underwent reoperation due to AL. Therefore, a multivariate analysis to detect independent risk factors for reoperation was not performed. Third, the final decision to create ileostomy was left to the surgeon, which may have introduced a selection bias. Finally, the timing of ileostomy closure was not standardized, which might have affected the surgical outcomes.

## Conclusions

In patients with CRC receiving resection surgery, tumor location in the lower rectum is associated with a high risk of AL. In such cases, ileostomy construction should be considered. Due to small number of AL patients requiring reoperation, the finding is exploratory. However, in patients with stenosis and T4 invasion, the merits of ileostomy might be restricted, and an alternative strategy, such as colostomy, might be required.

## Supplementary Information

Below is the link to the electronic supplementary material.

Supplementary file1 (DOCX 40 KB)

## Data Availability

The data that support the findings of this study are available on request from the corresponding author, Kohki Takeda.

## References

[1] Artus A, Tabchouri N, Iskander O, Michot N, Muller O, Giger-Pabst U et al (2020) Long term outcome of anastomotic leakage in patients undergoing low anterior resection for rectal cancer. BMC Cancer 20(1):780. 10.1186/s12885-020-07109-4 32819329 10.1186/s12885-020-07109-4 PMC7439541

[2] Boström P, Haapamäki MM, Rutegård J, Matthiessen P, Rutegård M (2019) Population-based cohort study of the impact on postoperative mortality of anastomotic leakage after anterior resection for rectal cancer. BJS Open 3(1):106–111. 10.1002/bjs5.50106 30734021 10.1002/bjs5.50106 PMC6354192

[3] Vinodkumar N, Khan ZA (2010) Multicentre analysis of oncological and survival outcomes following anastomotic leakage after rectal cancer surgery (Br J Surg 2009; 96: 1066–1075). Br J Surg 97(3):456–456. 10.1002/bjs.7009 20140945 10.1002/bjs.7009

[4] Mirnezami A, Mirnezami R, Chandrakumaran K, Sasapu K, Sagar P, Finan P (2011) Increased local recurrence and reduced survival from colorectal cancer following anastomotic leak: systematic review and meta-analysis. Ann Surg 253(5):890–899. 10.1097/SLA.0b013e3182128929 21394013 10.1097/SLA.0b013e3182128929

[5] Rahbari NN, Weitz J, Hohenberger W, Heald RJ, Moran B, Ulrich A et al (2010) Definition and grading of anastomotic leakage following anterior resection of the rectum: a proposal by the International Study Group of Rectal Cancer. Surgery 147(3):339–351. 10.1016/j.surg.2009.10.012 20004450 10.1016/j.surg.2009.10.012

[6] Garg PK, Goel A, Sharma S, Chishi N, Gaur MK (2019) Protective diversion stoma in low anterior resection for rectal cancer: a meta-analysis of randomized controlled trials. Visc Med 35(3):156–160. 10.1159/000497168 31367612 10.1159/000497168 PMC6616072

[7] Phan K, Kahlaee HR, Kim SH, Toh JWT (2019) Laparoscopic vs. robotic rectal cancer surgery and the effect on conversion rates: a meta-analysis of randomized controlled trials and propensity-score-matched studies. Tech Coloproctol 23(3):221–230. 10.1007/s10151-018-1920-0 30623315 10.1007/s10151-018-1920-0

[8] Güenaga KF, Lustosa SA, Saad SS, Saconato H, Matos D (2007) Ileostomy or colostomy for temporary decompression of colorectal anastomosis. Cochrane Database Syst Rev 2007(1):CD004647. 10.1002/14651858.CD004647.pub2 17253517 10.1002/14651858.CD004647.pub2 PMC8842962

[9] Rondelli F, Reboldi P, Rulli A, Barberini F, Guerrisi A, Izzo L et al (2009) Loop ileostomy versus loop colostomy for fecal diversion after colorectal or coloanal anastomosis: a meta-analysis. Int J Colorectal Dis 24(5):479–488. 10.1007/s00384-009-0662-x 19219439 10.1007/s00384-009-0662-x

[10] Geng HZ, Nasier D, Liu B, Gao H, Xu YK (2015) Meta-analysis of elective surgical complications related to defunctioning loop ileostomy compared with loop colostomy after low anterior resection for rectal carcinoma. Ann R Coll Surg Engl 97(7):494–501. 10.1308/003588415X14181254789240 26274752 10.1308/003588415X14181254789240 PMC5210131

[11] Tsujinaka S, Suzuki H, Miura T, Sato Y, Murata H, Endo Y et al (2023) Diagnosis, treatment, and prevention of ileostomy complications: an updated review. Cureus 15(1):e34289. 10.7759/cureus.34289 36721712 10.7759/cureus.34289 PMC9883118

[12] von Elm E, Altman DG, Egger M, Pocock SJ, Gøtzsche PC, Vandenbroucke JP (2007) The strengthening the reporting of observational studies in epidemiology (STROBE) statement: Guidelines for reporting observational studies. Ann Intern Med 147(8):573–577 17938396 10.7326/0003-4819-147-8-200710160-00010

[13] Elm E von, Altman DG, Egger M, Pocock SJ, Gøtzsche PC, Vandenbroucke JP et al (2025) The STROBE reporting checklist. In: Harwood J, Albury C, Beyer J de, Schlüssel M, Collins G (eds) The EQUATOR network reporting guideline platform [Internet]. The UK EQUATOR Centre. https://www.strobe-statement.org/checklists/

[14] Degiuli M, Elmore U, De Luca R, De Nardi P, Tomatis M, Biondi A et al (2022) Risk factors for anastomotic leakage after anterior resection for rectal cancer (RALAR study): a nationwide retrospective study of the Italian Society of Surgical Oncology Colorectal Cancer Network Collaborative Group. Colorectal Dis 24(3):264–276. 10.1111/codi.15997 34816571 10.1111/codi.15997 PMC9300066

[15] Zhang W, Lou Z, Liu Q, Meng R, Gong H, Hao L et al (2017) Multicenter analysis of risk factors for anastomotic leakage after middle and low rectal cancer resection without diverting stoma: a retrospective study of 319 consecutive patients. Int J Colorectal Dis 32(10):1431–1437. 10.1007/s00384-017-2875-8 28766076 10.1007/s00384-017-2875-8

[16] Platell C, Barwood N, Dorfmann G, Makin G (2007) The incidence of anastomotic leaks in patients undergoing colorectal surgery. Colorectal Dis 9(1):71–79. 10.1111/j.1463-1318.2006.01002.x 17181849 10.1111/j.1463-1318.2006.01002.x

[17] Kingham TP, Pachter HL (2009) Colonic anastomotic leak: risk factors, diagnosis, and treatment. J Am Coll Surg 208(2):269–278. 10.1016/j.jamcollsurg.2008.10.015 19228539 10.1016/j.jamcollsurg.2008.10.015

[18] Clausen FB, Dohrn N, Hölmich ER, Klein M, Gögenur I (2021) Safety of early ileostomy closure: a systematic review and meta-analysis of randomized controlled trials. Int J Colorectal Dis 36(2):203–212. 10.1007/s00384-020-03761-1 32970178 10.1007/s00384-020-03761-1

[19] Arenas Villafranca JJ, López-Rodríguez C, Abilés J, Rivera R, Gándara Adán N, Utrilla Navarro P (2015) Protocol for the detection and nutritional management of high-output stomas. Nutr J 14:45. 10.1186/s12937-015-0034-z 25956387 10.1186/s12937-015-0034-z PMC4461994

[20] Baker ML, Williams RN, Nightingale JM (2011) Causes and management of a high-output stoma. Colorectal Dis 13(2):191–197. 10.1111/j.1463-1318.2009.02107.x 19888956 10.1111/j.1463-1318.2009.02107.x

[21] Nakamura Y, Matsuda K, Yokoyama S, Hotta T, Takifuji K, Yamamoto M et al (2021) Intraoperative maneuvers may affect the development of early postoperative small bowel obstruction after laparoscopic colorectal cancer surgery: multicenter prospective cohort study. Int J Surg 86:52–56. 10.1016/j.ijsu.2021.01.007 33508470 10.1016/j.ijsu.2021.01.007

[22] Suwa K, Okamoto T, Yanaga K (2018) Risk factors for early postoperative small bowel obstruction after anterior resection for rectal cancer: methodological issues: reply. World J Surg 42(6):1908. 10.1007/s00268-018-4470-8 29344687 10.1007/s00268-018-4470-8

[23] Wang FG, Yan WM, Yan M, Song MM (2019) Comparison of anastomotic leakage rate and reoperation rate between transanal tube placement and defunctioning stoma after anterior resection: a network meta-analysis of clinical data. Eur J Surg Oncol 45(8):1301–1309. 10.1016/j.ejso.2019.01.182 30738589 10.1016/j.ejso.2019.01.182

